# Cognitive Behavioral Therapy for Anxiety in Parkinsonʼs Disease: A Randomized Controlled Trial

**DOI:** 10.1002/mds.28533

**Published:** 2021-02-22

**Authors:** Anja J.H. Moonen, Anne E.P. Mulders, Luc Defebvre, Annelien Duits, Bérengère Flinois, Sebastian Köhler, Mark L. Kuijf, Anne‐Claire Leterme, Dominique Servant, Marjolein de Vugt, Kathy Dujardin, Albert F.G. Leentjens

**Affiliations:** ^1^ Department of Psychiatry and Neuropsychology Maastricht University Medical Centre Maastricht the Netherlands; ^2^ Research School of Mental Health and Neuroscience (Mhens) Maastricht University Maastricht the Netherlands; ^3^ Neurology and Movement Disorders Department Centre Hospitalier Universitaire Lille France; ^4^ Department of Neurology Maastricht University Medical Centre Maastricht the Netherlands; ^5^ Degenerative & Vascular Cognitive Disorders University of Lille Lille France

**Keywords:** Parkinsonʼs disease, anxiety, randomized controlled trial, cognitive behavioral therapy

## Abstract

**Background:**

Anxiety disorders are among the most prevalent and disabling neuropsychiatric syndromes in patients with Parkinsonʼs disease (PD), but no randomized controlled treatment trials of anxiety have been published to date.

**Objective:**

The aim of this study was to assess the effectiveness of cognitive behavioral therapy (CBT) in the treatment of anxiety in patients with PD.

**Methods:**

Forty‐eight patients with PD with anxiety were randomized 1:1 between CBT and clinical monitoring only (CMO). The CBT program was developed to specifically address anxiety symptoms in PD and consisted of 10 weekly sessions. Assessments were conducted by blinded assessors at baseline, at the end of the intervention, after 3 months, and after 6 months (CBT group only). Main outcome measures were the Hamilton Anxiety Rating Scale (HARS) and the Parkinson Anxiety Scale (PAS).

**Results:**

Both the CBT and CMO groups showed clinically relevant improvement. Although there was no between‐group difference in outcome on the Hamilton Anxiety Rating Scale (6.7‐point reduction in the CBT group versus 3.9‐point reduction in the CMO group; *P* = 0.15), there was both a statistically significant and a clinically relevant between‐group difference on the total PAS in favor of CBT (9.9‐point reduction in the CBT group versus 5.2‐point reduction in the CMO group; *P* = 0.012), which was due to improvement on the PAS subscales for episodic (situational) anxiety and avoidance behavior. This greater improvement was maintained at 3‐ and 6‐month follow‐ups.

**Conclusion:**

CBT is an effective treatment for anxiety in patients with PD and reduces situational and social anxiety, as well as avoidance behavior. © 2021 The Authors. *Movement Disorders* published by Wiley Periodicals LLC on behalf of International Parkinson and Movement Disorder Society

Although Parkinson's disease (PD) is classically known as a motor disorder characterized by rest tremor, bradykinesia, rigidity, and postural instability, at least one‐third of patients also suffer from anxiety disorders.^1^ These are strongly associated with the severity of motor symptoms, a reduced quality of life, and an increased disability and mortality.^1^, ^2^ Anxiety in PD is likely the result of a complex interplay between pathophysiological and psychological factors. Due to its phenomenological overlap with depression, autonomic dysfunction, and other PD‐related somatic symptoms, such as wearing‐off, it is often difficult to recognize.^3^ As a result, anxiety in PD is widely underdiagnosed and untreated.^2^ Although the most commonly used treatment for anxiety in PD is pharmacological treatment with antidepressants and benzodiazepines, there have been no randomized, placebo‐controlled, clinical trials to test the efficacy of these medications.^4^ Moreover, the use of benzodiazepines is associated with undesirable side effects, such as reduced cognitive function, balance problems, and sedation, which may increase the risk of falls.^5^ Given this risk of side effects, there is a need for nonpharmacological psychotherapeutic interventions for the treatment of anxiety in PD.^4^ Cognitive behavioral therapy (CBT) is the most commonly used psychotherapy for anxiety in the general population.^6^ In PD, CBT has been proved effective for the treatment of depression, as well as impulse control disorders,^7^, ^8^ but there are as yet no randomized controlled trials (RCTs) on the effectiveness of CBT for anxiety. Uncontrolled studies have shown variable results, which is probably due to differences in procedures (eg, group versus individual therapy, face‐to‐face versus internet‐based therapy), small sample sizes, and enrollment of patients with mixed symptoms of depression and anxiety.^4^, ^9^, ^10^, ^11^, ^12^, ^13^

The aim of the present study was to assess the clinical effectiveness of a CBT program specifically tailored to treat anxiety in PD in a multicenter RCT. We hypothesized that CBT is more effective than clinical monitoring only (CMO) in reducing the level of anxiety symptoms.

## Patients and Methods

### Study Design

This study is a single‐blinded RCT in which patients with PD with anxiety were 1:1 randomized to an intervention group, receiving CBT plus clinical monitoring, or a control group, receiving CMO. All participants underwent a standardized clinical, cognitive, and behavioral assessment at baseline (t0), at the end of the intervention period (t1), as well as 3 months after the intervention (t2). The duration of the intervention period was approximately 10–12 weeks. In addition, participants randomized to the CBT group received a follow‐up assessment at 6 months (t3). Patients randomized to the CMO group did not receive this last assessment because it was not considered ethically acceptable to deny the control group routine psychotherapeutic or pharmacological treatment for a period longer than 6 months after the start of the intervention.

Written informed consent was obtained before participation and according to the guidelines of the Declaration of Helsinki. This study was approved by the medical ethics committees of both centers (Maastricht: METC azM/UM, NL56176.068.16; Lille: CPP Nord‐Ouest IV, 2016‐A 00966‐45). The trial was registered before inclusion of the first patient (ClinicalTrials.gov Identifier: NCT01792843), and its design was published.^14^

### Participants

Patients were recruited among outpatients of the movement disorders clinics of Maastricht University Medical Centre, the Netherlands, and Lille University Hospital, France.

Recruitment took place between January 2017 and March 2019, with the final follow‐up assessments taking place in September 2019. For details of the recruitment procedure, see the Supporting Information. Patients with idiopathic PD according to the Queens Square Brain Bank diagnostic criteria were included irrespective of their disease stage or current antiparkinsonian medication if they showed the presence of clinically relevant anxiety symptoms, as operationalized by a Parkinson Anxiety Scale (PAS) persistent (subscale A) score >9 and/or PAS avoidance (subscale C) score >3.^14^, ^15^ Comorbid depressive symptoms were not a reason for exclusion, as long as the patient did not meet the *Diagnostic and Statistical Manual of Mental Disorders*, Fifth Edition (DSM‐5) criteria for major depressive disorder. Pharmacotherapy for PD had to be stable for at least 1 month. Adjustments of antiparkinson medication during the trial were allowed if deemed clinically necessary. Pharmacotherapy for anxiety, for instance, with a selective serotonin reuptake inhibitor (SSRI), was allowed if the patient was receiving a stable dose for at least 2 months before inclusion and still met the inclusion criteria. Initiation or change of dosage of any psychopharmacological drug was reason for exclusion, as was psychotherapy. Medication use and use of mental health care outside the study were checked during each visit. For a full overview of inclusion and exclusion criteria, see Supporting Information Table S1. Inclusion criteria for the caregiver, either partner, family member, or informal caregiver, were: (1) daily contact with the study participant; and (2) no severe medical or psychiatric conditions, as determined by a clinical interview.

Three months after the start of the study, due to lagging inclusion, the inclusion criteria were adjusted: the availability of a caregiver and participation in the magnetic resonance imaging scanning part of the study were no longer required.

### Randomization and Masking

Baseline assessment was conducted after checking eligibility criteria and signing the informed consent form. Subsequently, participants were randomly assigned to CBT or CMO by the principal investigator (A.‐C.L.) using a randomized block design with 6 blocks of 10 patients each, generated through the website www.randomization.com (this website is presently retired, but still accessible as www.jerrydallal.com/random/randomize.htm). All follow‐up assessments were performed by a psychologist who was not involved in the intervention and was blinded for group allocation. Participants were instructed not to reveal any information on their group allocation. Blinding was abrogated for the 6‐month follow‐up assessment, because only participants in the intervention group received this assessment.

### Assessments

All patients underwent a full assessment at t0, t1, t2, and t3 (CBT group only). At baseline, demographic and disease‐related variables were recorded. The patient's medication was checked, and doses of antiparkinson medication were converted to levodopa equivalent daily dose.^15^ Caregivers were asked to complete questionnaires related to health‐related quality of life, well‐being, and health‐related costs (not reported in this article), as well as a questionnaire about caregiver burden. Primary outcome was the Hamilton Anxiety Rating Scale (HARS).^16^ The main secondary outcome was the PAS.^17^ See Table 1 for a full overview of the questionnaires administered.

**TABLE 1:** Questionnaires administered at t0, t1, t2, and t3

Domain	Instrument
Anxiety (primary outcome)	Hamilton Anxiety Rating Scale^16^
Anxiety (secondary outcomes)	Parkinson Anxiety Scale^17^
	Liebowitz Social Anxiety Scale^18^
	Mini International Neuropsychiatric Inventory section G and O^19^
Global cognitive status	Montreal Cognitive Assessment^20^
Depression	Hamilton Depression Rating Scale^21^
Apathy	Lille Apathy Rating Scale^22^
Sleep and nocturnal issues	Parkinsonʼs Disease Sleep Scale 2^23^
Coping strategies	Brief Cope scale^24^ Thought Control Questionnaire^25^
Motor symptom severity	MDS‐UPDRS^26^
PD disease stage	Hoehn & Yahr staging^27^
Caregiver burden	Zarit burden interview^28^
Health‐related quality of life	Parkinsonʼs disease Quality of Life Scale^29^

Abbreviations: t0, baseline; t1, end of the intervention period; t2, 3 months after the intervention; t3, follow‐up assessment at 6 months; MDS‐UPDRS, Movement Disorder Society Unified Parkinsonʼs Disease Rating Scale; PD, Parkinson's disease.

### Interventions

#### Cognitive Behavioral Therapy

The CBT program was specifically developed for the treatment of anxiety in PD. Based on focus group meetings with patients and their caregivers, the general format of existing CBT protocols for anxiety and depression was adjusted to better serve the needs and concerns of patients with PD. Specific aspects of anxiety in patients with PD, such as wearing‐off anxiety, fear of falling, feelings of shame, as well as comorbid neuropsychiatric and cognitive symptoms, were addressed (see Mulders et al.^14^ for more information). The complete CBT program consisting of a treatment manual for clinicians, as well as a full workbook for patients, will be available upon request in three different languages: English, French, and Dutch.

Patients received 10 weekly standardized individual sessions of 60–75 minutes. Several topics of anxiety were integrated with a specific focus on behavior and thoughts associated with anxiety. Any comorbid neuropsychiatric symptoms that were present, such as depressive symptoms or apathy, were addressed as well, although the main focus was on anxiety. Session topics included psychoeducation about anxiety, anxiety monitoring, self‐management (including sleep hygiene), relaxation techniques, thought restructuring, problem solving, exposure, and the development of a self‐management plan patients can use after closure of treatment. In addition, each patient received a booster session 6 weeks after the final treatment session to recall parts of the theory or exercises if necessary and to encourage continued use of the acquired skills and techniques.

Patients received a workbook during the first session, supplemented with handouts and worksheets that corresponded to the topic of each session. New home assignments were practiced during the session, and patients were being encouraged to practice daily the assignments at home. Any barriers or concerns for completing the home assignments were discussed at the end of each session. Involved caregivers were asked to be present during certain sessions and to provide motivational support at home if necessary.

All therapists responsible for conducting the CBT sessions (A.E.P.M., A.J.H.M., B.F., A.‐C.L.) were registered psychologists with ample experience with CBT. In each center, these psychologists were under supervision of a registered Cognitive Behavioral Therapist (A.D., M.d.V., D.S.), and therapy sessions were regularly discussed and evaluated.

#### Clinical Monitoring Only

All patients received clinical monitoring, either in addition to CBT or as sole treatment. Clinical monitoring involved providing general education leaflets on coping with anxiety, downloaded from the websites of the Netherlands' and the French psychiatric associations. In addition, 1 month after the baseline assessment, the participants were contacted by phone by an independent psychologist to inquire about current anxiety symptoms and any initiation or change of dosage of any psychopharmacological drug, as well as initiation of other psychological treatment, which would be reason for exclusion. Participants in the control group remained under the care of their treating physicians, who also monitored their medical and psychiatric status. Patients randomized to the control group were given the opportunity to receive CBT after the 3‐month follow‐up assessment.

### Sample Size Calculation

Power calculation was based on a standardized difference of 0.8 in HARS total score, with alpha set at 0.05 and power set at 0.80, and a predicted effect size of Cohen's d (0.95), based on a previous RCT with CBT for *depression* in PD.^7^ Based on these assumptions, the required sample size would be 40 (20 per group). Anticipating a 30% dropout during therapy, we decided to aim for a sample size of 60 patients (30 per group). The dropped‐out participants would not be replaced but included in the analysis according to the intention‐to‐treat (ITT) principle. A more detailed description of the power and sample size calculation can be found elsewhere.^14^

### Statistical Analysis

Analyses were performed with IBM SPSS statistics 24.0 and Stata 16. The numerical variables are described as means, median, standard deviations, and ranges. Categorical variables are described as frequencies and between‐group differences compared by chi‐square tests. Normality of the continuous variables was checked with the Kolmogorov–Smirnov test. Between‐group comparison of normally distributed variables was done by *t* tests; nonnormal distributed variables were compared by Mann–Whitney *U* test.

For the primary and secondary outcome variables, both within‐group and between‐group comparisons at baseline and different follow‐up times were analyzed using linear mixed model regression analyses. Likelihood ratio testing suggests that models with a random intercept had best fit. The ITT principle was taken into account by including all participants as randomized in the analyses, including dropouts, with maximum likelihood estimation of missing values. The level of significance, *P*, was set at 0.05 (two‐sided). The Benjamini–Hochberg correction was used to account for multiple comparisons. Effect sizes are reported as Cohen's d. In addition, a responder analysis was performed, in which response was defined as a reduction on the HARS or PAS score of at least 50% compared with baseline. The Fisher's exact test was used to compare proportions of responders.

## Results

### Participants

**FIG. 1:**
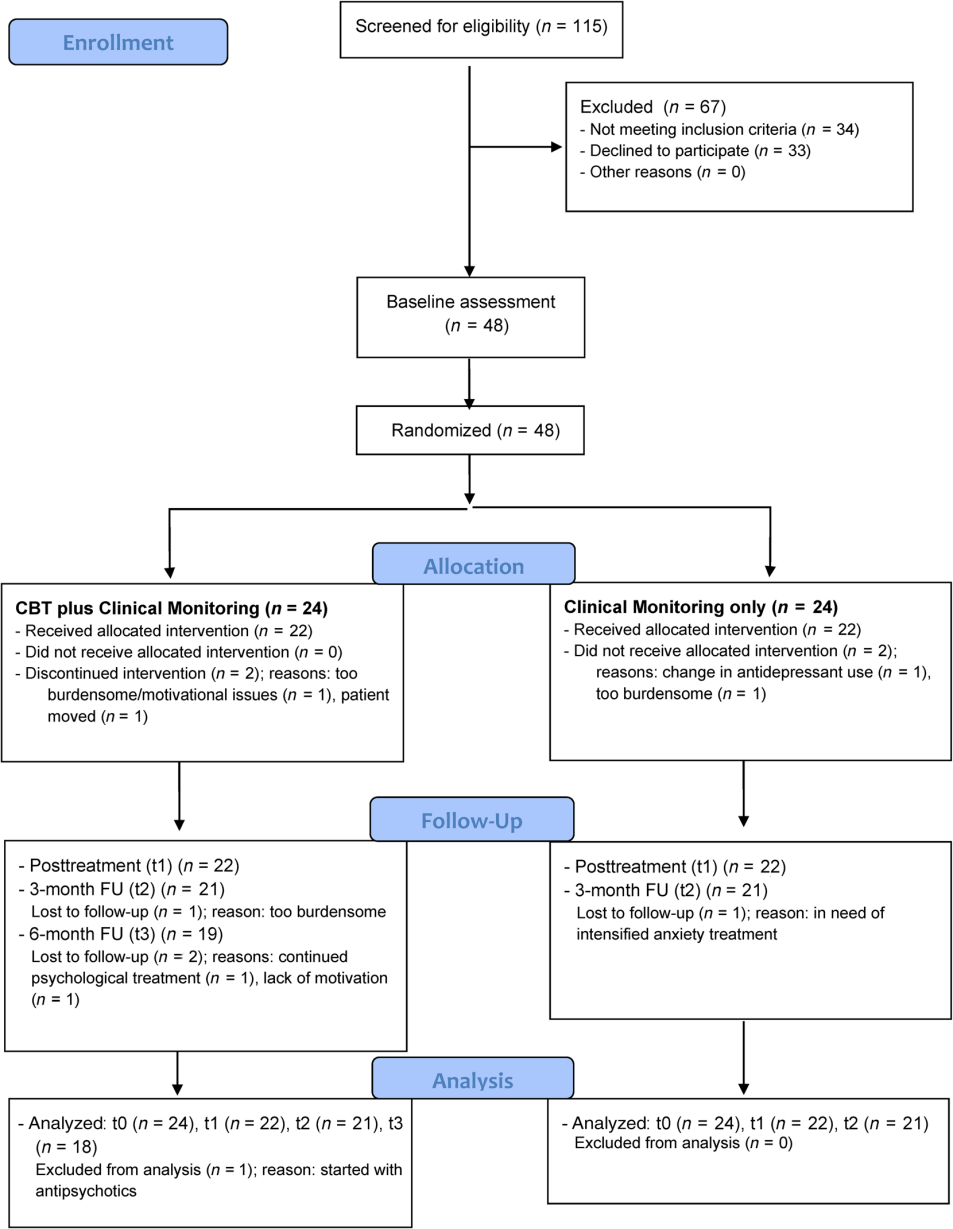
CONSORT flowchart for screening, inclusion, and dropout. CBT, cognitive behavioral therapy; FU, follow‐up. [Color figure can be viewed at wileyonlinelibrary.com]

Forty‐eight patients with PD were enrolled in the study, of which 24 patients were randomly assigned to the intervention group (CBT plus clinical monitoring) and 24 patients were assigned to the control group (CMO). Forty‐four patients (92%) completed the study period (t1), 42 patients (88%) additionally completed the 3‐month follow‐up (t2), and 19 patients in the intervention group (79%) completed the 6‐month follow‐up period (t3). There was no difference in dropouts or losses to follow‐up between the groups at 3 months: in each group, two patients (8.3%) dropped out during the intervention period and one patient (4.2%) during the follow‐up period. One patient was lost to the 6‐month follow‐up because of a hospital admission due to psychosis, which was thought to be unrelated to the CBT. For 14 patients in the intervention group (58%) and for 17 patients in the control group (71%), a caregiver was available during the intervention period. Numbers and reasons for dropout during the study period are presented in a ‘consolidated standards of reporting trials' (CONSORT) flow diagram in Fig. 1. Due to the slow inclusion and the less than expected dropout, the inclusion of patients was stopped before the target of 60 patients because the minimal required sample size was reached.

### Baseline Characteristics

Twenty‐five women and 23 men with an average age of 63.3 [standard deviation (SD) 7.8] years were included. The average disease duration was 6.1 (SD 5.0) years, the median Hoehn and Yahr (H&Y) was stage 2 (range 1–3), and the average score on section 3 (motor) of the Movement Disorder Society Unified Parkinsonʼs Disease Rating Scale (MDS‐UPDRS) was 26.4 (SD 12.3). All patients had clinically relevant anxiety symptoms with an average score of 18.4 (SD 4.7) on the HARS and 23.3 (SD 6.4) on the PAS, with scores indicating clinically relevant anxiety on the subscales for persistent anxiety (PAS‐A), episodic anxiety (PAS‐B), and avoidance behavior (PAS‐C). Baseline demographic and clinical characteristics are presented in Table 2 and Supporting Information Table S2. Only the score on the PAS‐B was not normally distributed. There were no statistically significant differences in any of the demographic and clinical measures, nor in outcome measures at baseline between the two groups. There was a trend for a higher score on the total PAS and the PAS subscales for episodic anxiety, as well as avoidance behavior in the CBT group. Although this did not reach statistical significance, it was considered a clinically relevant confounder. Therefore, subsequent analyses were corrected for baseline differences on these three scales.

**TABLE 2:** Baseline demographic and clinical characteristics of the included patients

	Total sample (n = 48)	CBT (n = 24)	CMO (n = 24)	*P* value
Center, n (%)				
Maastricht	19 (40)	9 (38)	10 (42)	
Lille	29 (60)	15 (63)	14 (58)	
Sex, n (%)				0.77
Male	23 (48)	11 (46)	12 (50)	
Female	25 (52)	13 (54)	12 (50)	
Marital status, n (%)				0.60
Never married	0 (0)	0 (0)	0 (0)	
Married/living in couple	41 (85)	20 (83)	21 (88)	
Widowed	1 (4)	1 (4)	0 (0)	
Divorced/separated	6 (26)	3 (13)	3 (13)	
Age, yr, mean (SD)	63.3 (7.8)	63.3 (7.2)	63.3 (8.4)	1.00
Education, yr, mean (SD)	13.8 (3.7)	13.2 (3.4)	14.4 (3.8)	0.26
Parkinsonʼs disease duration, yr, mean (SD)	6.1 (5.0)	7.4 (5.6)	4.7 (4.0)	0.06
Age of Parkinsonʼs disease onset, yr, mean (SD)	57.4 (7.9)	56.0 (6.8)	58.9 (8.7)	0.21
Levodopa equivalent daily dose, mg, mean (SD)	800.1 (800.3)	839.7 (1018.6)	760.5 (517.7)	0.74
Hoehn & Yahr stage (median)	2 (r 1–3)^a^	2 (r 1–3)^a^	2 (r 1–3)^a^	0.60
MDS‐UPDRS part 2: EDL score (r 0–52), mean (SD)	11.9 (5.9)	11.7 (5.8)	13.1 (5.9)	0.17
MDS‐UPDRS part 3: motor score (r 1–132), mean (SD)	26.4 (12.3)	25.7 (13.7)	27.2 (9.9)	0.53
Current DSM‐5 diagnosis				
Social phobia disorder, n (%)	19 (40)	8 (33)	11 (46)	0.38
Generalized anxiety disorder, n (%)	34 (71)	15 (63)	19 (80)	0.20
Comorbid depressive disorder^a^, n (%)	7 (15)	3 (13)	4 (17)	0.68
Levodopa use, n (%)	40 (83)	20 (83)	20 (83)	1.00
Dopamine‐agonist use, n (%)	19 (40)	10 (42)	9 (38)	0.77
Antidepressant use, n (%)	9 (19)	4 (17)	5 (21)	0.71
Benzodiazepine use, n (%)	9 (19)	3 (13)	6 (25)	0.27
Antipsychotics use, n (%)	0 (0)	0 (0)	0 (0)	–
History of anxiety disorder, n (%)	10 (21)	6 (25)	4 (17)	0.54
History of depression, n (%)	18 (38)	9 (38)	9 (38)	0.17
History of panic attacks, n (%)	21 (44)	13 (54)	8 (33)	0.32
History of obsessive‐compulsive disorder, n (%)	3 (6)	1 (4)	2 (8)	0.55
Family history of Parkinsonʼs disease, n (%)	11 (23)	5 (21)	6 (25)	0.73
Family history of anxiety disorder, n (%)	8 (17)	5 (21)	3 (13)	0.44
Family history of depressive disorder, n (%)	9 (19)	5 (21)	4 (17)	0.71
Caregiver involved, n (%)	31 (65)	14 (58)	17 (71)	0.37
**Outcome measures (theoretical range)**				
Primary				
Hamilton Anxiety Rating Scale score (0–52), mean (SD)	18.4 (4.7)	18.4 (4.4)	18.3 (5.1)	0.976
Secondary				
Parkinson Anxiety Scale total score (0–52), mean (SD)	23.3 (6.4)	25.0 (6.7)	21.7 (6.0)	0.07
Parkinson Anxiety Scale subscale A persistent anxiety (0–24), mean (SD)	13.2 (0.4)	13.3 (0.5)	13.1 (0.6)	0.83
Parkinson Anxiety Scale subscale B episodic anxiety (0–16), mean (SD)	5.6 (0.5)	6.4 (0.7)	4.8 (0.5)	0.05
Parkinson Anxiety Scale subscale C avoidance behavior (0–12), mean (SD)	4.5 (0.4)	5.3 (0.6)	3.8 (0.5)	0.08
Liebowitz Social Anxiety Scale score (0–144), mean (SD)	45.3 (24.7)	48.6 (27.8)	42.0 (21.1)	0.36
Hamilton Depression Rating Scale score (0–50), mean (SD)	11.0 (4.3)	11.8 (4.2)	10.3 (4.5)	0.23
Lille Apathy Rating Scale score (−36 to +36), mean (SD)	−25.2 (6.6)	−25.3 (5.9)	−25.0 (7.4)	0.88
Parkinsonʼs Disease Questionnaire‐8 score (0–32), mean (SD)	11.8 (4.4)	11.8 (4.9)	11.8 (3.9)	0.95

^a^ As defined by a HAMD score >16.

Abbreviations: CBT, cognitive behavioral therapy; CMO, clinical monitoring only; SD, standard deviation; r, range; analyses from chi‐square tests and independent samples *t* test, except for subscale B of the Parkinson Anxiety Scale: Mann–Whitney *U* test; MDS‐UPDRS, Movement Disorder Society Unified Parkinsonʼs Disease Rating Scale; EDL, Experiences of Daily Living; DSM, *Diagnostic and Statistical Manual of Mental Disorders* of the American Psychiatric Association.

### Quantitative Treatment Effects Over Time

#### Primary Outcome

**FIG. 2:**
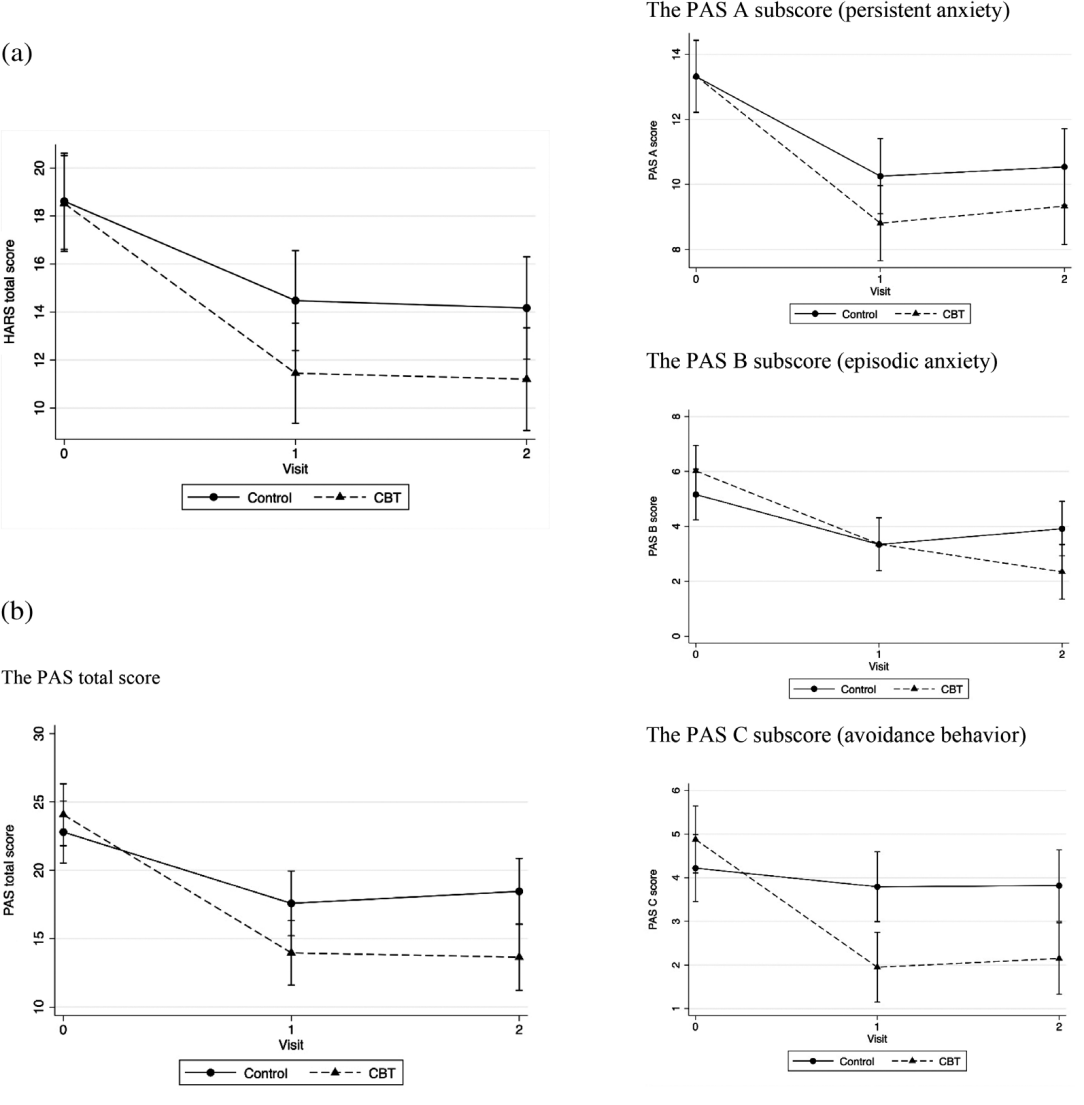
Graphic representation of treatment effects. (A) Improvement on the Hamilton Anxiety Rating Scale (HARS). (B) Improvement on the total Parkinson Anxiety Scale (PAS) and PAS subscales: the PAS total score, the PAS‐A subscore (persistent anxiety), the PAS‐B subscore (episodic anxiety), and the PAS‐C subscore (avoidance behavior).

At the end of the intervention period (t1), the mean HARS score in the intervention group improved by 6.7 points from 18.4 (SD 4.4) to 11.7 (SD 7.1), while in the control group it improved 3.9 points from 18.3 (SD 5.1) to 14.4 (SD 5.3). Whereas the within‐group improvement was statistically significant for both groups (*P* < 0.001), the difference in improvement between the groups was not (*P* = 0.15) (Table 3 and Fig. 2A). The within‐group improvements were still present in both arms at the 3‐month follow‐up (*P* < 0.001) and at 6‐month follow‐up for the CBT group (*P* < 0.001).

**TABLE 3:** Time by treatment effects in the CBT and CMO groups, with statistical comparison

	CBT, mean (SD)	CMO, mean (SD)	*z* ^a^	*P* value	Effect size
**Primary**					
Hamilton Anxiety Rating Scale					
Baseline	18.4 (4.4)	18.3 (5.1)	−0.07	0.95	
Posttreatment	11.7 (7.1)	14.4 (5.3)	−1.50	0.14	0.57
3‐mo follow‐up	11.7 (5.8)	14.1 (7.2)	−1.44	0.15	0.50
6‐mo follow‐up	12.0 (5.9)			**<0.001**	
**Secondary**					
Parkinson Anxiety Scale					
Baseline	25.0 (6.7)	21.7 (6.0)	1.27	0.44	
Posttreatment	15.1 (8.3)	16.5 (6.2)	−4.89	**0.03**	0.74
3‐mo follow‐up	15.0 (7.8)	17.4 (7.2)	−6.08	**0.006**	0.89
6‐mo follow‐up	14.3 (7.0)			**<0.001**	
Parkinson Anxiety Scale subscale A persistent					
Baseline	13.3 (2.5)	13.1 (2.9)	0.02	0.73	
Posttreatment	8.9 (4.2)	10.3 (4.0)	−1.45	0.15	4.0
3‐mo follow‐up	9.5 (4.2)	10.5 (4.0)	−1.19	0.23	3.2
6‐mo follow‐up	8.7 (3.8)			**<0.001**	
Parkinson Anxiety Scale subscale B episodic^b^					
Baseline	6.4 (3.5)	4.8 (2.7)	1.28	0.20	
Posttreatment	3.8 (3.0)	2.8 (2.5)	−0.88	0.38	1.4
3‐mo follow‐up	2.9 (2.4)	3.4 (2.6)	−2.50	**0.01**	4.7
6‐mo follow‐up	3.3 (2.5)			**<0.001**	
Parkinson Anxiety Scale subscale C avoidance^b^					
Baseline	5.3 (3.0)	3.8 (2.6)	1.18	0.24	
Posttreatment	2.3 (1.9)	3.4 (2.2)	−3.15	**0.002**	6.17
3‐mo follow‐up	2.6 (2.9)	3.5 (2.3)	−2.90	**0.004**	5.74
6‐mo follow‐up	2.3 (2.3)			**<0.001**	
Liebowitz Social Anxiety Scale					
Baseline	48.6 (27.8)	42.0 (21.1)	0.20	0.84	
Posttreatment	36.7 (24.5)	48.4 (28.0)	−2.71	**0.007**	0.47
3‐mo follow‐up	38.4 (26.0)	45.2 (30.2)	−1.94	**0.05**	0.28
6‐mo follow‐up	35.1 (24.1)			**0.001**	
Hamilton Depression Rating Scale					
Baseline	11.8 (4.1)	10.3 (4.5)	0.34	0.73	
Posttreatment	7.7 (5.4)	9.9 (5.5)	−2.86	**0.004**	0.50
3‐mo follow‐up	8.6 (4.8)	9.3 (4.3)	−1.89	0.06	0.15
6‐mo follow‐up	8.9 (4.6)			**0.015**	
Lille Apathy Rating Scale score					
Baseline	−25.3 (5.9)	−25.0 (7.4)	0.02	0.98	
Posttreatment	−27.4 (7.1)	−22.1 (8.5)	−2.43	**0.02**	0.79
3‐mo follow‐up	−27.1 (6.2)	−24.4 (7.0)	−0.98	0.33	0.41
6‐mo follow‐up (n = 17)	−26.2 (8.8)			0.67	
Parkinsonʼs Disease Questionnaire‐8					
Baseline	11.8 (4.9)	11.8 (3.9)	−0.01	0.99	
Posttreatment	9.32 (5.5)	10.5 (4.4)	−0.73	0.47	0.26
3‐mo follow‐up	9.62 (5.3)	10.8 (5.4)	−0.94	0.35	0.08
6‐mo follow‐up	10.22 (4.1)			0.12	
Zarit Burden Interview (caregivers)					
Baseline	9.9 (6.6)	11.5 (6.6)			
Posttreatment	8.2 (6.3)	12.4 (7.5)	−1.70	0.09	
3‐mo follow‐up	10.8 (5.1)	11.2 (8.6)	0.54	0.59	
6‐mo follow‐up	13.5 (5.9)				

Abbreviations: CBT, cognitive behavioral therapy; CMO, clinical monitoring only; SD, standard deviation.

^a^ *z*‐Statistic for linear mixed model regression analyses; *z* scores and *P* values listed in the table pertain to change from baseline. Effect sizes are listed as Cohen's d. For all analyses: df = 133; for the 6‐mo follow‐up, within‐group statistics (change from baseline) are reported, because only patients in the CBT group had a 6‐mo follow‐up.

^b^ Corrected for baseline differences.

#### Secondary Outcomes

If the PAS is taken as the outcome measure, apart from within‐group improvements in both groups at all assessment times (all *P* < 0.001) (Supporting Information Table S2), there were also clear and significant between‐groups posttreatment differences in favor of CBT (*P* = 0.026). In the CBT group, the score on the PAS declined by 9.9 points from 25 (SD 6.7) to 15.1 (SD 8.3) and in the CMO group by 5.2 points from 21.7 (SD 6.0) to 16.5 (SD 6.2) (Table 3 and Fig. 2B). On the subscales of the PAS, there were significant between‐group differences in favor of CBT for episodic anxiety at 3‐month follow‐up (*P* = 0.012) and avoidance behavior at the end of treatment and 3‐month follow‐up (*P* = 0.004) (see Table 3 and Fig. 2B). For episodic anxiety, the initial improvement in the CMO group is lost at follow‐up, whereas the improvement in the CBT group has increased. For the intervention group, these improvements were maintained at 6‐month follow‐up (*P* < 0.001) (Supporting Information Table S2). There was no significant between‐group difference in score on the PAS subscale for persistent anxiety. Social anxiety as measured with the Liebowitz Social Anxiety Scale also showed a clear improvement, with a between‐group difference favoring CBT (*P* = 0.007), which is still marginally significant at 3‐month follow‐up (*P* = 0.052). In the intervention group, the 6‐month follow‐up shows a better score again, compared with the 3‐month follow‐up (*P* = 0.001). After the intervention, there was a significantly greater improvement in mood, measured with the Hamilton Depression Rating Scale (HAMD), in the CBT groups compared with the CMO group (*P* = 0.004), which still showed a clear trend at 3‐month follow‐up (*P* = 0.058). In the CBT group, the improvement was maintained at 6‐month follow‐up. For apathy, as measured with the Lille Apathy Rating Scale (LARS), there is a between‐group difference in favor of CBT at the end of the intervention that loses significance at 3‐month follow‐up. There is no improvement in quality of life as measured with the Parkinson's Disease Quality of Life Scale (PDQ‐8).^29^ Caregivers did not report a lesser burden of care on the Zarit Burden Interview.^28^ For an overview of the most important outcome measures, see Table 3. There were no between‐group differences in other outcomes (Supporting Information Table S3).

### Responder Analyses

The proportion of responders was not significantly different between patients randomized to CBT or CMO. On the HARS, 9 of 21 patients (43%) receiving CBT responded versus 5 of 20 patients (25%) receiving CMO (*P* = 0.33). On the PAS, 6 of 21 patients (29%) in the CBT group responded versus 4 of 20 (20%) in the CMO group (*P* = 0.72) (Supporting Information Table S4). On at the PAS subscales, only subscale C (avoidance behavior) shows a significant between‐group difference (*P* = 0.03), with 13 of 21 patients (62%) in the CBT group responding versus 5 of 19 (26%) in the CMO group.

## Discussion

This is the first RCT of the effectiveness of a CBT treatment program specifically tailored to treat anxiety in patients with PD. Both the CBT and the CMO groups showed improvements in anxiety. On the primary outcome, the HARS, there was no between‐group difference in improvement. However, on the secondary outcome, the PAS, there was a significant and clinically relevant between‐group difference, with effect sizes ranging from 1.4 to 4.7 on the PAS subscales for episodic anxiety and avoidance behavior. In the responder analysis, only the subscale for avoidance behavior showed a significant between‐group difference. We chose the HARS, rather than the PAS, as the primary outcome measure because this is the standard in anxiety research and because at the time the study protocol was conceived, no information on sensitivity to change was available for the PAS. In retrospect, the PAS would have been a better outcome measure. Nine of 13 of the HARS items refer to physical symptoms that may also be attributable to the motor symptoms and nonmotor symptoms of PD other than anxiety, whereas the PAS was designed to be insensitive to motor symptom severity and comorbid depressive symptoms.^17^ In this study, the PAS turned out to be more sensitive to change than the HARS. Looking at the PAS subscales, this improvement was due to improvements for episodic anxiety and avoidance behavior, which are the areas that are specifically addressed by CBT. Episodic anxiety in this context does not only refer to panic but also to situational anxiety, such as fear of falling, wearing‐off anxiety, among others. Such situational anxiety, as well as avoidance behaviors, is characteristic for anxiety in PD.^30^, ^31^ As such, our study confirms preliminary evidence from earlier open studies that also report a specific effect on fear of falling.^9^, ^13^ This is also supported by the significant between‐group difference in improvement of social anxiety as measured with the Liebowitz Social Anxiety Scale. Moreover, treatment effects are lasting and sustained at 3‐month and 6‐month follow‐ups. We conclude that the PAS is a better measure to monitor treatment progress in patients with PD than the HARS. The improvement of anxiety in patients randomized to CMO could be seen as a benefit of the minimal intervention of providing information on anxiety and a phone call, which may also have increased a placebo response. Also, participating in the screening visit and baseline assessment may have made patients more aware of their symptoms and that these can in fact be improved.

Although we designed our module specifically for anxiety and not for depression, CBT does have a prolonged effect on mood, which is absent in the CMO group. There are no sustained effects on apathy and quality of life. The latter is in contrast with the positive feedback received by patients who received CBT. Because it is to be expected that reducing situational anxiety, as well as avoidance behavior, would contribute to an increased quality of life, we think that the items of the PDQ‐8 are not specific and sensitive enough to adequately measure subjective well‐being and experienced quality of life as a result of anxiety.

Strong points in this study are the randomized controlled design and the fact that assessors were blind to the group assignment. Also, the program was specifically designed, in collaboration with patients and caregivers, to treat anxiety in patients with PD. It was well accepted and dropout was low: 9% in both the CBT and CMO groups, which is much lower than the average dropout of 20% reported in CBT studies for anxiety in patients not suffering from PD, even after motivational interviewing.^32^ Limitations are the fact that the role of “nonspecific factors” that contribute to the effectiveness of therapy, such as the motivation and dedication of the therapists, cannot be quantified. Also, whereas the module was tailored to address anxiety in patients with PD, it was at the same time standardized and thus not entirely tailored to address specific aspects of anxiety in individual patients. The effectiveness of CBT may be even larger if there is freedom to adjust the module to individual patient's needs. Moreover, it is also not possible to identify which parts of the CBT program were most helpful. The included patients were in mild‐to‐moderate disease stages without relevant cognitive decline, without relevant psychiatric comorbidity, and able and willing to come to the clinic for treatment. It is unknown whether the results can be generalized to more severely affected patients with cognitive decline or other psychiatric morbidity. Adjustments of antiparkinson medication were allowed if considered clinically necessary and, in those cases, may have affected anxiety symptoms as well. Other potential limitations are the fact that there was only one booster session, and follow‐up was limited to 3 months for the CMO group and to 6 months for the CBT group. Long‐term effects of CBT, as well as the effect of additional booster sessions cannot be assessed.

## Conclusion

CBT is an effective treatment for anxiety in patients with PD and mainly reduces episodic anxiety, avoidance behavior, and social anxiety. The program can be further optimized to address individual patient's needs. Future research should also focus on improving cost‐effectiveness and improving access to treatment by addressing alternative ways of administering CBT, such as by group therapy or internet‐based therapy.

## Financial Disclosures

L. Defebvre gave consultancies to AbbVie and Orkyn and earned honoraria for lectures from AbbVie and UCB.

A.F.G. Leentjens received a research grant from the Michael J. Fox Foundation and earned royalties from “Springer media” and “de Tijdstroom.”

All other authors have nothing to disclose.

## Author Roles

1. Research project: A. Conception, B. Organization, C. Execution.

2. Statistical Analysis: A. Design, B. Execution, C. Review and Critique.

3. Manuscript Preparation: A. Writing of the first draft, B. Review and Critique.

A.J.H.M.: 1A, 1B, 1C, 3A.

A.E.P.M.: 1B, 1C, 2B, 3B.

L.D.: 2C, 3B.

A.D.: 1B, 1C, 2B.

B.F.: 1C, 3B.

S.K.: 2A, 2B, 2C, 3B.

M.L.K.: 2C, 3B.

A.‐C.L.: 1C, 3B.

D.S.: 1C, 3B.

M.d.V.: 1C, 3B.

K.D.: 1A, 1B, 1C, 2C, 3C.

A.F.G.L.: 1A, 1B, 1C, 2A, 2B, 3B.

## Supporting information


Supporting Information

## References

[1] Broen MP , Narayen NE , Kuijf ML , Dissanayaka NN , Leentjens AF . Prevalence of anxiety in Parkinson's disease: a systematic review and meta‐analysis. Mov Disord 2016;31:1125–1133. 27125963 10.1002/mds.26643

[2] Dissanayaka NN , White E , O'Sullivan JD , Marsh R , Silburn PA , Copland DA , et al. Characteristics and treatment of anxiety disorders in Parkinson's disease. Mov Disord Clin Pract 2015;2:155–162. 30363816 10.1002/mdc3.12157 PMC6183244

[3] Rutten S , Ghielen I , Vriend C , Hoogendoorn AW , Berendse HW , Leentjens AF , et al. Anxiety in Parkinson's disease: symptom dimensions and overlap with depression and autonomic failure. Parkinsonism Relat Disord 2015;21:189–193. 25557888 10.1016/j.parkreldis.2014.11.019

[4] Pontone GM , Dissanayka N , Apostolova L , Brown RG , Dobkin R , Dujardin K , et al. Report from a multidisciplinary meeting on anxiety as a non‐motor manifestation of Parkinson's disease. NPJ Parkinsons Dis 2019;5:30. 31840044 10.1038/s41531-019-0102-8 PMC6906437

[5] Bloem BR , Grimbergen YA , Cramer M , Willemsen M , Zwinderman AH . Prospective assessment of falls in Parkinson's disease. J Neurol 2001;248:950–958. 11757958 10.1007/s004150170047

[6] Cuijpers P , Sijbrandij M , Koole S , Huibers M , Berking M , Andersson G . Psychological treatment of generalized anxiety disorder: a meta‐analysis. Clin Psychol Rev 2014;34:130–140. 24487344 10.1016/j.cpr.2014.01.002

[7] Dobkin RD , Menza M , Allen LA , Gara MA , Mark MH , Tiu J , et al. Cognitive‐behavioral therapy for depression in Parkinson's disease: a randomized, controlled trial. Am J Psychiatry 2011;168:1066–1074. 21676990 10.1176/appi.ajp.2011.10111669 PMC3186855

[8] Okai D , Askey‐Jones S , Samuel M , O'Sullivan SS , Chaudhuri KR , Martin A , et al. Trial of CBT for impulse control behaviors affecting Parkinson patients and their caregivers. Neurology 2013;80:792–799. 23325911 10.1212/WNL.0b013e3182840678 PMC3598451

[9] Reynolds GO , Saint‐Hilaire M , Thomas CA , Barlow DH , Cronin‐Golomb A . Cognitive‐behavioral therapy for anxiety in Parkinson's disease. Behav Modif 2020;44(4):552–579. 30931594 10.1177/0145445519838828 PMC8137685

[10] Koychev I , Okai D . Cognitive‐behavioural therapy for non‐motor symptoms of Parkinson's disease: a clinical review. Evid Based Ment Health 2017;20:15–20. 28073810 10.1136/eb-2016-102574 PMC10688422

[11] Calleo JS , Amspoker AB , Sarwar AI , Kunik ME , Jankovic J , Marsh L , et al. A pilot study of a cognitive‐behavioral treatment for anxiety and depression in patients with Parkinson disease. J Geriatr Psychiatry Neurol 2015;28:210–217. 26047635 10.1177/0891988715588831

[12] Kraepelien M , Svenningsson P , Lindefors N , Kaldo V . Internet‐based cognitive behavioral therapy for depression and anxiety in Parkinson's disease ‐ a pilot study. Internet Interv 2015;2:1–6.

[13] Dissanayaka NNW , Pye D , Mitchell LK , Byrne GJ , O'Sullivan JD , Marsh R , et al. Cognitive behavior therapy for anxiety in Parkinson's disease: outcomes for patients and caregivers. Clin Gerontol 2017;40:159–171. 28452666 10.1080/07317115.2016.1240131

[14] Mulders AEP , Moonen AJH , Dujardin K , Kuijf ML , Duits A , Flinois B , et al. Cognitive behavioural therapy for anxiety disorders in Parkinson's disease: design of a randomised controlled trial to assess clinical effectiveness and changes in cerebral connectivity. J Psychosom Res 2018;112:32–39. 30097133 10.1016/j.jpsychores.2018.04.002

[15] Tomlinson CL , Stowe R , Patel S , Rick C , Gray R , Clarke CE . Systematic review of levodopa dose equivalency reporting in Parkinson's disease. Mov Disord 2010;25:2649–2653. 21069833 10.1002/mds.23429

[16] Hamilton M . The assessment of anxiety states by rating. Br J Med Psychol 1959;32:50–55. 13638508 10.1111/j.2044-8341.1959.tb00467.x

[17] Leentjens AF , Dujardin K , Pontone GM , Starkstein SE , Weintraub D , Martinez‐Martin P . The Parkinson Anxiety Scale (PAS): development and validation of a new anxiety scale. Mov Disord 2014;29:1035–1043. 24862344 10.1002/mds.25919

[18] Liebowitz MR . Social phobia. Mod Probl Pharmacopsychiatry 1987;22:141–173. 2885745 10.1159/000414022

[19] Sheehan DV , Lecrubier Y , Sheehan KH , Amorim P , Janavs J , Weiller E , et al. The Mini‐International Neuropsychiatric Interview (M.I.N.I.): the development and validation of a structured diagnostic psychiatric interview for DSM‐IV and ICD‐10. J Clin Psychiatry 1998;59(suppl 20):22–33. 9881538

[20] Nasreddine ZS , Phillips NA , Bédirian V , Charbonneau S , Whitehead V , Collin I , et al. The Montreal Cognitive Assessment, MoCA: a brief screening tool for mild cognitive impairment. J Am Geriatr Soc 2005;53:695–699. 15817019 10.1111/j.1532-5415.2005.53221.x

[21] Hamilton M . A rating scale for depression. J Neurol Neurosurg Psychiatry 1960b;23:56–62. 14399272 10.1136/jnnp.23.1.56 PMC495331

[22] Sockeel P , Dujardin K , Devos D , Deneve C , Destee A , Defebvre L . The Lille apathy rating scale (LARS), a new instrument for detecting and quantifying apathy: validation in Parkinson's disease. J Neurol Neurosurg Psychiatry 2006;77:579–584. 16614016 10.1136/jnnp.2005.075929 PMC2117430

[23] Trenkwalder C , Kohnen R , Hogl B , Metta V , Sixel‐Doring F , Frauscher B , et al. Parkinson's disease sleep scale‐validation of the revised version PDSS‐2. Mov Disord 2011;26:644–652. 21312275 10.1002/mds.23476

[24] Carver CS . You want to measure coping but your protocol's too long: consider the brief COPE. Int J Behav Med 1997;4:92–100. 16250744 10.1207/s15327558ijbm0401_6

[25] Wells A , Davies MI . The thought control questionnaire ‐ a measure of individual‐differences in the control of unwanted thoughts. Behav Res Ther 1994;32(8):871–878. 7993332 10.1016/0005-7967(94)90168-6

[26] Goetz CG , Tilley BC , Shaftman SR , Stebbins GT , Fahn S , Martinez‐Martin P , et al. Movement Disorder Society‐sponsored revision of the Unified Parkinson's Disease Rating Scale (MDS‐UPDRS): scale presentation and clinimetric testing results. Mov Disord 2008;23:2129–2170. 19025984 10.1002/mds.22340

[27] Hoehn MM , Yahr MD . Parkinsonism: onset, progression and mortality. Neurology 1967;17:427–442. 6067254 10.1212/wnl.17.5.427

[28] Zarit SH , Reever KE , Bach‐Peterson J . Relatives of the impaired elderly: correlates of feelings of burden. Gerontologist 1980;20:649–655. 7203086 10.1093/geront/20.6.649

[29] Jenkinson C , Fitzpatrick R , Peto V , Greenhall R , Hyman N . The PDQ‐8: development and validation of a short‐form Parkinson's disease questionnaire. Psychol Health 1997;12:805–814.

[30] Broen MPG , Leentjens AFG , Hinkle JT , Moonen AJH , Kuijf ML , Fischer NM , et al. Clinical markers of anxiety subtypes in Parkinson disease. J Geriatr Psychiatry Neurol 2018;31:55–62. 29528763 10.1177/0891988718757369 PMC5903060

[31] Perepezko K , Hinkle JT , Shepard MD , Fischer N , Broen MPG , Leentjens AFG , et al. Social role functioning in Parkinson's disease: a mixed‐methods systematic review. Int J Geriatr Psychiatry 2019;34:1128–1138. 31069845 10.1002/gps.5137 PMC6949188

[32] Marker I , Norton PJ . The efficacy of incorporating motivational interviewing to cognitive behavior therapy for anxiety disorders: a review and meta‐analysis. Clin Psychol Rev 2018;62:1–10. 29727863 10.1016/j.cpr.2018.04.004

