# Supplementary material for: Cognitive Behavioral Therapy for Anxiety in Parkinsonʼs Disease: A Randomized Controlled Trial
Source: Mov Disord. 2021 Feb 22;36(11):2539–48. doi: 10.1002/mds.28533 (PMC9290129; doi:10.1002/mds.28533)
Supplement: Supplementary file 1 — Supporting Information [file MDS-36-2539-s001.docx]

Supplementary material to Moonen et al.: Cognitive behavioral therapy for anxiety in Parkinson’s disease: a randomized controlled trial

**Recruitment procedure**

During routine outpatient visits, patients were informed about the study and asked whether they would allow giving their contact information to the investigators. In that they were given the study information including the informed consent form. They were told that the investigator would call the patient to answer any questions. Two weeks after this call, the patient would be called again to answer additional questions and to ask for their decision whether or not to participate. Informed consent was signed in site in the presence of the investigator, so the patient would have the opportunity to ask additional questions. The participants received a copy of this signed form.

| **Table S1.** In- and exclusion criteria for Parkinson’s disease (PD) patients |
| --- |
| **Inclusion criteria** |
| - Idiopathic PD according to the Queens Square Brain Bank diagnostic criteria (14) |
| - Presence of clinically relevant anxiety symptoms, as operationalized by a Parkinson Anxiety Scale (PAS) persistent score > 9 and/or PAS avoidance score > 3 (15) |
| - Using a stable dose of antiparkinsonian medication for at least 1 month |
| - No other current psychological treatment for anxiety |
| - Age between 35 and 80 years old - Presence of a caregiver* - Willingness to undergo MRI scanning pre- and post-treatment* |
| **Exclusion criteria** |
| - Parkinsonian syndromes or neurodegenerative disorders other than PD |
| - Dementia or severe cognitive decline, operationalized as a Montreal Cognitive Assessment (MoCa) score < 24 (16) |
| - Major depressive disorder (MDD) as defined by the criteria of a DSM 5 diagnosis for MDD (17) |
| - Abuse of alcohol, drugs or benzodiazepines |
| Abbreviations: PD = Parkinson’s disease, PAS = Parkinson Anxiety Scale, MoCa = Montreal Cognitive Assessment, MDD = Major Depressive Disorder; DSM 5= Diagnostic and Statistical Manual of mental disorders version, 5^th^ edition.  * These criteria were dropped after three months to facilitate inclusion. |

**Table S2. Additional outcome variables at baseline of the included patients**

|  |  | **total sample** | **CBT** | **CMO** | **p** |
| --- | --- | --- | --- | --- | --- |
|  | **theoretical score range** | **mean (SD)** | **mean (SD)** | **mean (SD)** |  |
|  |  |  |  |  |  |
| Montreal Cognitive Assessment score | 0-30 | 26.3 (2.4) | 26.6 (2.1) | 26.1 (2.7) | 0.48 |
| Thought Control Questionnaire score | 30-120 | 60.7 (10.9) | 61.1 (11.4) | 60.3 (10.6) | 0.81 |
| Parkinson’s Disease Sleep Scale-2 score | 0-60 | 17.9 (8.1) | 18.6 (8.6) | 17.3 (7.7) | 0.58 |
| Brief-COPE emotion-focused scale score* | 10-40 | 21.1 (4.5) | 20.5 (4.4) | 21.7 (4.6) | 0.39 |
| Brief-COPE problem-focused scale score* | 6-24 | 14.5 (3.7) | 14.3 (3.5) | 14.8 (3.9) | 0.64 |
| Brief-COPE dysfunctional scale score* | 1 | 22.4 (3.8) | 22.0 (3.3) | 22.9 (4.3) | 0.43 |
| Zarit Burden Interview (caregivers) | 0-48 | 10.8 (6.6) | 9.9 (6.6) | 11.5 (6.6) | 0.51 |

CBT = cognitive behavioral therapy, CMO= clinical monitoring only, SD= standard deviation

* The division of the COPE in three subsections was based on Cooper et al.

The emotion-focused subscale consists of items 3, 7, 9, 14, 16, 17, 18, 19, 20 and 21.

The problem-focused subscale consists of items 1, 2, 6, 10, 26 and 28.

The dysfunctional subscale consists of items 4, 5, 8, 11, 12, 13, 15, 22, 23, 24, 25 and 27

Reference: Cooper C, Katona C, Orrell M, et al. [Coping strategies and anxiety in caregivers of people with Alzheimer's disease: the LASER-AD study.](https://pubmed.ncbi.nlm.nih.gov/16337688/)  J Affect Disord. 2006;90:15-20.

**Table S3. Between group outcomes for additional measures**

|  | **CBT** | **CMO** |  |  |
| --- | --- | --- | --- | --- |
|  | **Mean (SD)** | **Mean (SD)** | **z*** | **p** |
|  |  |  |  |  |
| Thought Control Questionnaire |  |  |  |  |
| Baseline | 61.1 (11.4) | 60.3 (10.6) | 0.14 | 0.89 |
| Posttreatment | 60.9 (9.4) | 58.4 (8.5) | 0.75 | 0.46 |
| 3-months follow-up | 64.2 (7.4) | 62.2 (12.0) | 0.59 | 0.56 |
| 6-months follow-up | 61.6 (10.1) |  | 0.01 | 0.99 |
|  |  |  |  |  |
| Parkinson’s Disease Sleep Scale-2 score |  |  |  |  |
| Baseline | 18.6 (8.6) | 17.3 (7.7) | 0.22 | 0.83 |
| Posttreatment | 17.1 (10.1) | 17.0 (7.0) | -0.48 | 0.63 |
| 3-months follow-up | 18.9 (9.3) | 17.6 (6.7) | 0.07 | 0.94 |
| 6-months follow-up | 19.1 (8.8) |  | 0.76 | 0.45 |
| Brief-COPE emotion-focused scale score# |  |  |  |  |
| Baseline | 20.5 (4.4) | 21.7 (4.6) | -0.23 | 0.82 |
| Posttreatment | 22.1 (5.0) | 20.7 (5.3) | 2.14 | **0.03** |
| 3-months follow-up | 21.6 (4.7) | 21.9 (4.9) | 0.89 | 0.38 |
| 6-months follow-up | 21.3 (5.5) |  | 0.52 | 0.64 |
|  |  |  |  |  |
| Brief-COPE problem-focused scale score# |  |  |  |  |
| Baseline | 14.3 (3.5) | 14.8 (3.9) | -0.14 | 0.89 |
| Posttreatment | 15.6 (2.9) | 15.0 (3.7) | 0.79 | 0.43 |
| 3-months follow-up | 15.1 (3.6) | 14.9 (5.3) | 0.33 | 0.74 |
| 6-months follow-up | 14.8 (4.9) |  | 0.73 | 0.47 |
|  |  |  |  |  |
| Brief-COPE dysfunctional scale score# |  |  |  |  |
| Baseline | 22.0 (3.3) | 22.9 (4.3) | -0.27 | 0.79 |
| Posttreatment | 22.4 (3.6) | 23.4 (5.0) | -0.04 | 0.97 |
| 3-months follow-up | 22.4 (4.7) | 22.5 (4.4) | 0.67 | 0.51 |
| 6-months follow-up | 22.3 (3.6) |  | 0.45 | 0.69 |
|  |  |  |  |  |
| MDS-UPDRS part 2 (ADL) |  |  |  |  |
| Baseline | 10.7 (5.8) | 13.1 (5.9) | 0.03 | 0.97 |
| Posttreatment | 9.8 (5.2) | 12.9 (7.5) | -0.31 | 0.75 |
| 3-months follow-up | 22.0 (12.5) | 13.8 (7.9) | 0.80 | 0.42 |
| 6-months follow-up | 10.7 (4.7) |  | 0.40 | 0.69 |
|  |  |  |  |  |
| MDS-UPDRS part 3 (motor) |  |  |  |  |
| Baseline | 25.7 (13.7) | 27.2 (9.9) | -0.14 | 0.89 |
| Posttreatment | 23.7 (14.2) | 32.2 (11.6) | -2.88 | **0.004** |
| 3-months follow-up | 22.0 (12.5) | 29.9 (10.3) | -2.67 | **0.008** |
| 6-months follow-up (N=17) | 22.9 (11.8) |  | -1.56 | 0.118 |

CBT = cognitive behavioral therapy, CM= clinical monitoring, SD= standard deviation

* = z-statistic for linear mixed model regression analyses; z-scores and p-values listed in the table pertain to change from baseline. Effect sizes refer to Cohen’s d. For all analyses: df = 133; only patients in the intervention group had a 6-month follow-up.

# for subdivision of the COPE: see comments with supplementary table 1.

**Table S4. Post-treatment responder analysis**

| Outcome | Responder (n) | | non-responder (n) | | Fisher-exact p |
| --- | --- | --- | --- | --- | --- |
| Treatment | CBT n=21 | CMO n=20 | CBT  n=21 | CMO n=20 |  |
|  |  |  |  |  |  |
| primary outcome |  |  |  |  |  |
| HARS | 9 | 5 | 12 | 15 | 0.33 |
|  |  |  |  |  |  |
| secondary outcome |  |  |  |  |  |
| PAS total | 6 | 4 | 15 | 16 | 0.72 |
| - PAS-A (persistent) | 7 | 2 | 14 | 18 | 0.14 |
| - PAS-B (episodic)* | 9/20 | 8/19 | 11/20 | 11/19 | 1.00 |
| - PAS-C (avoidance)* | 13 | 5/19 | 8 | 14/19 | 0.03 |

* Sample size may actually be smaller in for these outcomes because 1 and 2 patients respectively did not have this subtype of anxiety (i.e. scored zero on these subscales) and hence have not been classified as responder or non-responder.
